# Machine learning for the development of diagnostic models of decompensated heart failure or exacerbation of chronic obstructive pulmonary disease

**DOI:** 10.1038/s41598-023-39329-6

**Published:** 2023-08-05

**Authors:** César Gálvez-Barrón, Carlos Pérez-López, Felipe Villar-Álvarez, Jesús Ribas, Francesc Formiga, David Chivite, Ramón Boixeda, Cristian Iborra, Alejandro Rodríguez-Molinero

**Affiliations:** 1 Research Area, Consorci Sanitari Alt Penedès i Garraf, Sant Pere de Ribes-Barcelona, Barcelona, Spain; 2 Department of Pneumology, IIS Fundación Jiménez Díaz, CIBERES, Madrid, Spain; 3 Department of Pneumology, Hospital Universitari de Bellvitge, Barcelona, Spain; 4 Geriatric Unit, Department of Internal Medicine, Hospital Universitari de Bellvitge, Barcelona, Spain; 5 Department of Internal Medicine, Hospital de Mataró, Mataró-Barcelona, Spain; 6 Department of Cardiology, IIS Fundación Jiménez Díaz, Madrid, Spain

**Keywords:** Cardiology, Diseases, Medical research

## Abstract

Heart failure (HF) and chronic obstructive pulmonary disease (COPD) are two chronic diseases with the greatest adverse impact on the general population, and early detection of their decompensation is an important objective. However, very few diagnostic models have achieved adequate diagnostic performance. The aim of this trial was to develop diagnostic models of decompensated heart failure or COPD exacerbation with machine learning techniques based on physiological parameters. A total of 135 patients hospitalized for decompensated heart failure and/or COPD exacerbation were recruited. Each patient underwent three evaluations: one in the decompensated phase (during hospital admission) and two more consecutively in the compensated phase (at home, 30 days after discharge). In each evaluation, heart rate (HR) and oxygen saturation (Ox) were recorded continuously (with a pulse oximeter) during a period of walking for 6 min, followed by a recovery period of 4 min. To develop the diagnostic models, predictive characteristics related to HR and Ox were initially selected through classification algorithms. Potential predictors included age, sex and baseline disease (heart failure or COPD). Next, diagnostic classification models (compensated vs. decompensated phase) were developed through different machine learning techniques. The diagnostic performance of the developed models was evaluated according to sensitivity (S), specificity (E) and accuracy (A). Data from 22 patients with decompensated heart failure, 25 with COPD exacerbation and 13 with both decompensated pathologies were included in the analyses. Of the 96 characteristics of HR and Ox initially evaluated, 19 were selected. Age, sex and baseline disease did not provide greater discriminative power to the models. The techniques with S and E values above 80% were the logistic regression (S: 80.83%; E: 86.25%; A: 83.61%) and support vector machine (S: 81.67%; E: 85%; A: 82.78%) techniques. The diagnostic models developed achieved good diagnostic performance for decompensated HF or COPD exacerbation. To our knowledge, this study is the first to report diagnostic models of decompensation potentially applicable to both COPD and HF patients. However, these results are preliminary and warrant further investigation to be confirmed.

## Introduction

Heart failure (HF) and chronic obstructive pulmonary disease (COPD) are two chronic diseases with the greatest adverse impact on the general population^1–3^. Decompensation (in HF) or exacerbation (in COPD) are especially important since they affect autonomy and quality of life and increase mortality and the need for hospital admission or visits to emergency services^4–7^. Therefore, developing methods that allow early detection of the decompensation of these diseases is important since such detection allows faster recovery and avoids the need for a major intervention such as hospital admission^8,9^.

The usual approach of the methods developed to date to detect early decompensation of both diseases is based on ambulatory monitoring of clinical parameters using predictive models or diagnostic algorithms applied continuously or intermittently^10,11^. Regarding HF, a systematic review of algorithms based on noninvasive physiological parameters^11^ identified the most frequently considered physiological parameters for this type of algorithm: weight as a marker of fluid overload (96%), blood pressure (85%), heart rate (HR) (61%), oxygen saturation (Ox) (23%), and heart rhythm (17%). However, the optimal combination of parameters for detecting decompensation has not yet been established, and body weight, although widely used, often generates many false alerts and has low sensitivity for detecting cardiac decompensation. In COPD, unlike in HF, the most commonly considered physiological parameters are Ox and HR^10^, as well as lung function tests (spirometry). Several studies^12–14^ have calculated the differences in these parameters in the days leading up to a COPD exacerbation. Thus, for Ox, a decrease of 1–2 points or 1–1.24 standard deviations (SDs) from the baseline has been reported, and for HR, elevations of 5–7 beats per minute or 3 SDs from the baseline have been reported. Among the aforementioned parameters for both conditions, we emphasized Ox and HR since they are physiological parameters that can currently be remotely and reliably monitored through nondisruptive technological devices during patients' daily routines.

Although various algorithms have been developed to date, very few overcome the sensitivity (S) and specificity (E) threshold of 80%^15–18^. In addition, some of those that overcome this barrier are based on monitoring from invasive devices implanted in patients (such as pacemakers or defibrillators)^15^ or the introduction of specific devices in their homes, such as indoor air quality analysers^17^, all of which restrict their widespread use.

Our group previously developed and reported diagnostic algorithms for the detection of COPD exacerbation (S: 90%, E: 89%) and decompensated HF (S: 85%, E: 75%) based on noninvasive monitoring of physiological parameters of patients (HR, Ox and walking distance) in compensated and decompensated phases of their diseases^19^. The “expert rules” algorithms were developed based on an analysis of the mean physiological parameters evaluated and a strategy including parallel and serial tests^20^. Despite the good diagnostic performance observed, these algorithms suffer from some important limitations, such as inefficient exploitation of the data (although the data were collected continuously second by second, the analysis was based on reducing these data to their means) and the absence of validation. These difficulties limit the acceptance and application of these algorithms in routine clinical practice.

To overcome these limitations, we believe that machine learning (ML) techniques can be useful. This approach allows more efficient and individualized use of the vast amount of data produced from continuous monitoring of physiological parameters^21^, especially with respect to early detection of risky clinical situations^21,22^. An individualized approach that allows this technique has been proposed as a tool to improve the limitation of false-positives, which frequently occur when fixed and identical limits or thresholds are used for all patients^22,23^. Likewise, the use of these techniques is increasingly frequent in regard to implementing algorithms for monitoring physiological parameters in “real conditions”^24^, which may avoid the need for controlled situations or specific protocols for the application of the developed algorithms. Finally, unlike the usual statistical techniques where inference is usually the most important factor (that is, investigation of the relationships between variables or understanding a phenomenon rather than its identification or detection), ML techniques have a primary purpose of prediction or identification of a situation or event (for example, to identify if a patient is in the decompensated phase of a chronic disease)^25^.

In this study, we report the diagnostic performance of diagnostic algorithms based on physiological parameters (HR and Ox) and developed with ML techniques to classify patients’ disease phases (compensated or decompensated). The recommended guidelines for reporting this type of study have been considered^26–28^.

## Materials and methods

### Design

This was a prospective multicenter observational study. Unlike studies on prognostic models, in the present study, diagnostic models were developed, that is, models designed to determine whether a patient was in the compensated or decompensated phase of their disease (exacerbation of COPD and/or HF decompensation).

### Sample

The criteria for admission to this study and the recruitment process have been previously reported^19^. Patients older than 55 years who were able to walk at least 30 m, with a main diagnosis of decompensated HF and/or exacerbation of COPD and hospitalized in the Department of Internal Medicine, Cardiology or Pneumology were included. Participants with a pacemaker or intracardiac device, domiciliary oxygen therapy users prior to admission and patients with HF functional class IV of the New York Heart Association (NYHA) classification were excluded^29^.

Four hospitals participated: two tertiary university hospitals (600–900 hospital beds) and two regional secondary care hospitals (150–400 hospital beds) in the provinces of Barcelona and Madrid.

Each center had a trained interviewer, and each department had a referring physician who was accessible to the interviewer. Each day, the interviewer contacted the referring physician to review the hospitalization census and identify patients with the diagnosis of interest. Next, the interviewer confirmed the main diagnosis (decompensated HF and/or exacerbation of COPD) with the physician responsible for the patient and then contacted the participant (the same day or the next day) to obtain informed consent and verify compliance with all admission criteria of this study. The sample was obtained through convenience sampling, and all patients were enrolled consecutively as they were identified.

The recruitment and follow-up periods lasted 18 months starting in November 2010.

### Evaluation of the participants

Each patient underwent three identical evaluations: the first in the hospitalization unit (V1) and the other two consecutively and at least 24 h apart in the participant’s home 30 days after hospital discharge (V2 and V3). Thus, each participant underwent one evaluation in the decompensated phase (V1) and two in the compensated phase (V2, V3) of their disease.

The evaluation protocol^19^ included documentation of symptoms (dyspnea according to the NYHA^29^ and Modified Medical Research Council (mMRC)^30^ scales) and physiological parameters (HR and Ox) in two consecutive periods: effort (walking at a normal pace and on flat terrain for a maximum of 6 min) and recovery (seated for 4 min after the end of the effort period).

HR and Ox were considered time series with a sample frequency of 1 Hz and were collected throughout the evaluation with a pulse oximeter (Model 3100, brand Nonin® Medical, Inc., Plymouth, MN, USA) placed on the left index finger.

### Reference standard diagnostic test

Given the absence of a single standard diagnostic test to verify whether a patient was in the compensated or decompensated phase of their disease, the clinical judgment of the participant’s responsible physician was considered a standard diagnostic test. Thus, in the decompensated phase, the diagnosis of decompensated HF and/or COPD exacerbation corresponded to the confirmed diagnosis from the participant’s attending physician (in cases of diagnostic doubt, the patient was excluded). For the compensated phase, a standard diagnosis of compensated HF and/or stable COPD was confirmed by a study physician through telephone contact with the participant 30 days after hospital discharge. During this telephone interaction, the patient was considered to be in the compensated phase if none of the following events had occurred since hospital discharge: increased cough, sputum or dyspnea; initiation of or an increase in corticosteroid use; and initiation of antibiotic treatment or medical consultation for worsening of the clinical situation from any cause. In cases of doubt or if the compensated phase could not be confirmed, successive telephone contacts were made until the phase could be confirmed. The interviewer scheduled home visits for the respective evaluations (V2, V3) only after confirmation and within 24–48 h of receiving confirmation.

### Index test: diagnostic algorithms

#### Initial preparation of potentially predictive variables or characteristics

Given the objective of this study (development of an “online” algorithm capable of detecting the onset of an exacerbation from HR and Ox data), various characteristics of each of the evaluations were extracted (V1, V2, V3). For this purpose, the effort phase (walking) and recovery phase of each evaluation were separated by verifying the times recorded manually in the data collection records at the beginning and end of each phase of the test and visually reviewing the signals to confirm the manual records. Once the signals were separated according to the evaluation phase, the corresponding characteristics of the available measures were extracted.

Numerous characteristics were extracted from the signals. During each of the tests, two different phases were considered: effort and recovery, which were treated separately. From each of the phases, three signals were considered: HR, Ox and the normalized difference between these variables. From each of these three temporal signals, the characteristics of the temporal (the mean, standard deviation, and range) and frequency domains (the characteristics of the first and second harmonics, the distribution of the harmonics [kurtosis and skewness], the sum of all harmonics and the six first indices of the principal component analysis [PCA] for the normalized fast Fourier transform [FFT] of the signal) were extracted. Accordingly, 16 characteristics were obtained from each phase (effort and recovery) of each signal (HR, Ox, and the normalized difference between these), resulting in a total of 96 characteristics for each evaluation. The normalized difference between Ox and HR was defined using the *sklearn standardscaler* function (the mathematical formula is available at https://scikit-learn.org/stable/modules/generated/sklearn.preprocessing.StandardScaler.html), and PCA was applied to the HR and Ox time series using the *sklearn.decomposition.PCA* function (formula available at https://scikit-learn.org/stable/modules/generated/sklearn.decomposition.PCA.html). Regarding the selection of the first 6 components of the PCA, this decision was made based on the researchers' criteria, considering that typically in this type of analysis, the first 3 to 6 components are considered.

#### Labeling and definition of events to be detected

**Figure 1:**
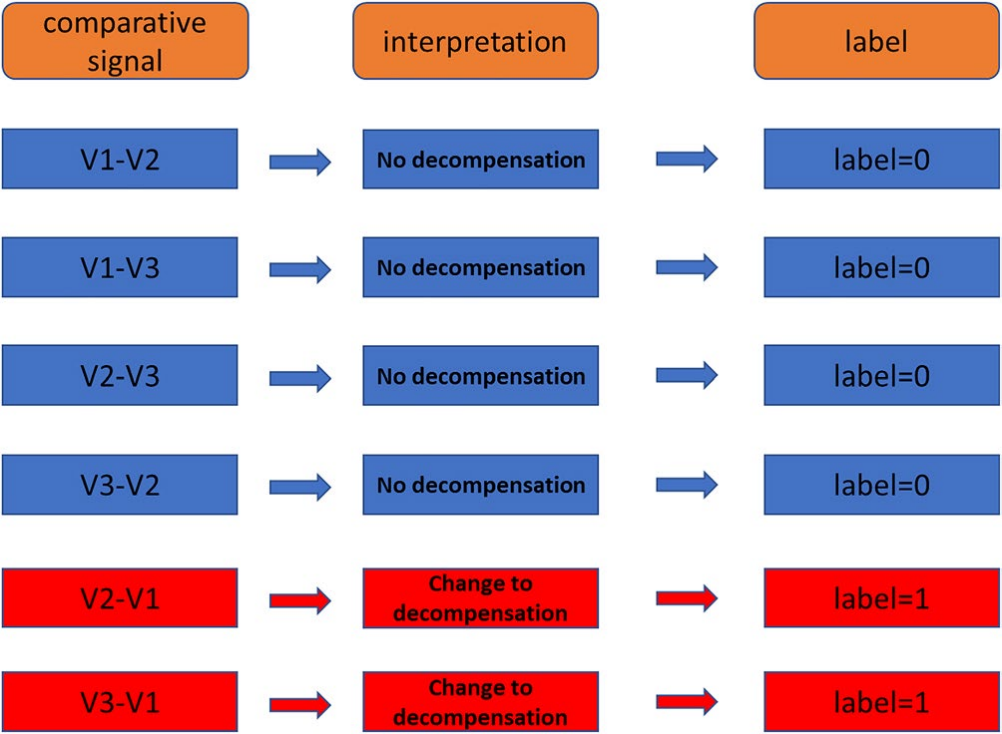
Labeling and interpretation of comparative signals.

Given that the main objective of this study was the detection of a transition from a state considered normal or stable (HF or COPD in the compensated phase [V2, V3]) to a state of decompensation or exacerbation (decompensated phase [V1]), a methodological scheme was applied based on calculation of the differences between the evaluations of each available characteristic. Thus, if a patient had three evaluations (V1, V2 and V3), six differences or useful comparative signals were obtained from these evaluations (V1–V2, V1–V3, V2–V1, V2–V3, V3–V1, V3–V2). The label of each of these comparative signals is illustrated in Fig. 1.

Although the differences V1–V2 and V1–V3 might be more appropriately considered “decompensation recovery” rather than “no decompensation”, we decided to discard a third label category (“decompensation recovery”) due to the small sample size and because the main objective of the trial was the detection of decompensation.

#### Selection of predictor variables or characteristics

In a first approximation, potential predictive characteristics were selected using the random forest^31^, gradient boosting classifier^31^ and light gradient-boosting machine (LGBM)^32^ classification algorithms, which integrate the functions of characteristic selection by importance within the decision. We selected the top 10 features based on their importance ranking within the structure of each classifier model.

**Figure 2:**
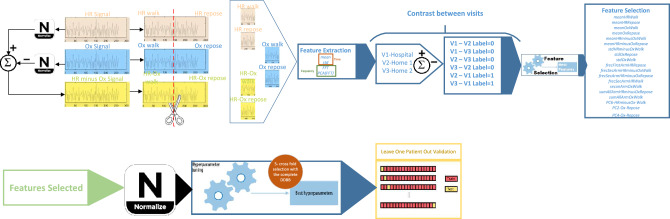
Process for preparation and selection of the characteristics of the evaluations.

Figure 2 shows an outline of the process for preparation and selection of the characteristics of the signals.

During the process of selecting characteristics, all those that were redundant or had very low variabilities were discarded. In this study, by definition, we did not have variables with perfect separation that could cause overestimation of the diagnostic capacity of the models (overfitting)^26^.

In addition to the characteristics selected from the HR and Ox signals, the age, sex and baseline disease (HF or COPD) of the patients were considered potential predictors.

### Development and validation of algorithms

For the development of the algorithms, the ML techniques most used in the studies of classification models were considered: (i) decision trees, (ii) random forest, (iii) k-nearest neighbor (KNN), (iv) support vector machine (SVM), (v) logistic regression, (vi) naive Bayes classifier, (vii) gradient-boosting classifier and (viii) LGBM.

For each of these techniques, hyperparameters were selected based on a brute force scheme using all available data through a cross-validation scheme (*K-fold cross-validation*, k = 5). A normalization process based on the medians and interquartile ranges (IQRs) was applied to all characteristics^31^.

**Figure 3:**
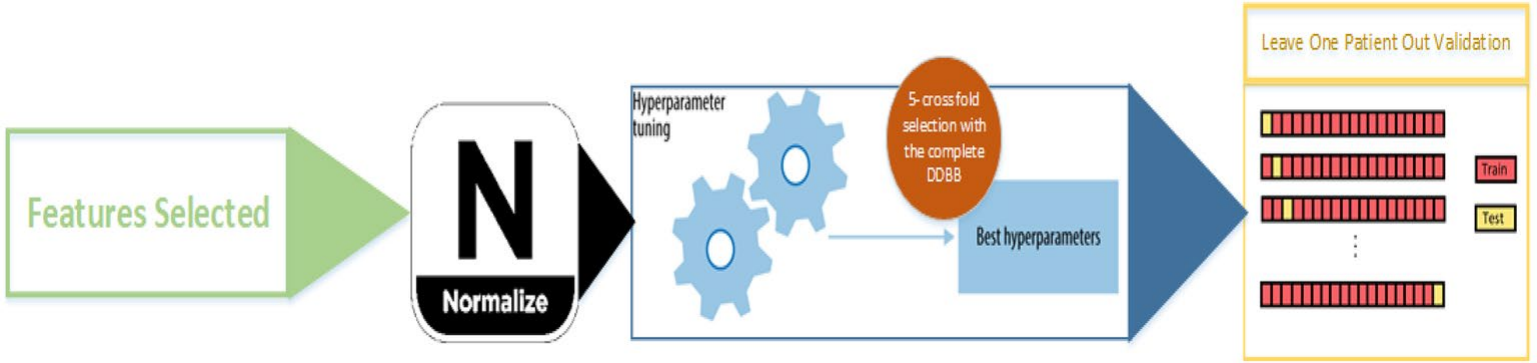
Scheme of the training and validation of the study algorithms.

Once the best parameters of each technique were identified, internal validation was performed with a leave-one-patient-out method. Thus, a new model was calculated for each patient by replacing the model’s data from the training and validation sets with the patient’s data. Figure 3 shows an outline of the training and validation process.

The observation units (inputs) on which the algorithms were applied were the differences between two different evaluations, as illustrated in Fig. 1. Thus, the algorithms classified the evaluated difference as a state of “no decompensation” (label = 0) or “a change to decompensation” (label = 1). Therefore, the following parameters were defined:

- *True positive (TP)* “a change to decompensation” as the classification result for a V3–V1 or V2–V1 comparison.
- *True negative (TN)* “no decompensation” as the classification result for a V1–V2, V1–V3, V2–V3 or V3–V2 comparison.
- *False positive (FP)* “change to decompensation” as the classification result for a V1–V2, V1–V3, V2–V3 or V3–V2 comparison.
- *False negative (FN)* “no decompensation” as the classification result for a V3–V1 or V2–V1 comparison.

The parameters used to evaluate the diagnostic performance of the algorithms were S, E and accuracy (A). Each patient could have up to six observation units or inputs; therefore, up to six classification results were obtained, which were then defined as TP, TN, FP or FN. Then, the S, E and A were obtained for each patient. The final S, E and A of the entire sample were calculated from the mean of the parameters obtained from each patient.

The predictive values were not considered because the proportions of evaluations in the decompensated phase (33% [V1]) and compensated phase (66% [V2, V3]) did not correspond to the usual proportion found in clinical practice (the vast majority of patients in the community are usually in the compensated phase).

### Missing data, excluded data and indeterminate results

Missing data were not included in the analysis, but patients with missing data were not excluded (all available patient data were included in the analysis). No imputation of the missing data was performed.

During the process of signal review and verification of the start and end times of each evaluation from the manual records, missing sections of HR and/or Ox data due to poor contact between the skin and the sensor were observed. This incidence caused the introduction of some filters to be applied to exclude these missing sections from the analysis. Thus, an evaluation was excluded if it had a loss rate (missing measures divided by the total number of measures) greater than 10% in any phase. In addition, evaluations performed at home (V2, V3) that did not reveal an improvement in the sensation of dyspnea for the patient (of at least one point according to the mMRC scale^30^) with respect to the decompensated phase evaluation (V1) were also excluded to ensure that home assessments were performed in the “compensated phase”.

No indeterminate results were noted in the index test (algorithms); in all cases, the model produced a “no decompensation” or “a change to decompensation” result. On the other hand, all evaluations were always performed after a definitive result of the standard diagnostic reference test: clinical diagnosis of the decompensated phase by the doctor responsible for the patient in the hospital evaluation (V1) and clinical diagnosis of the compensated phase by the doctor who contacted the patients by phone before home evaluations (V2, V3). Thus, the algorithms were developed and applied on evaluations clearly labeled as the compensated or decompensated phase by the reference diagnostic test.

### Ethics committee approval

All methods and procedures were performed in accordance with the relevant guidelines and regulations. The study followed the principles contained in the Declaration of Helsinki and approved by the Ethics and Research Committee (ERC) of the center promoting the study (ERC of the Mataró Hospital, approval number 1851806). Informed consent was obtained from all participants and/or their legal guardians.

## Results

### Participants and evaluations

**Figure 4:**
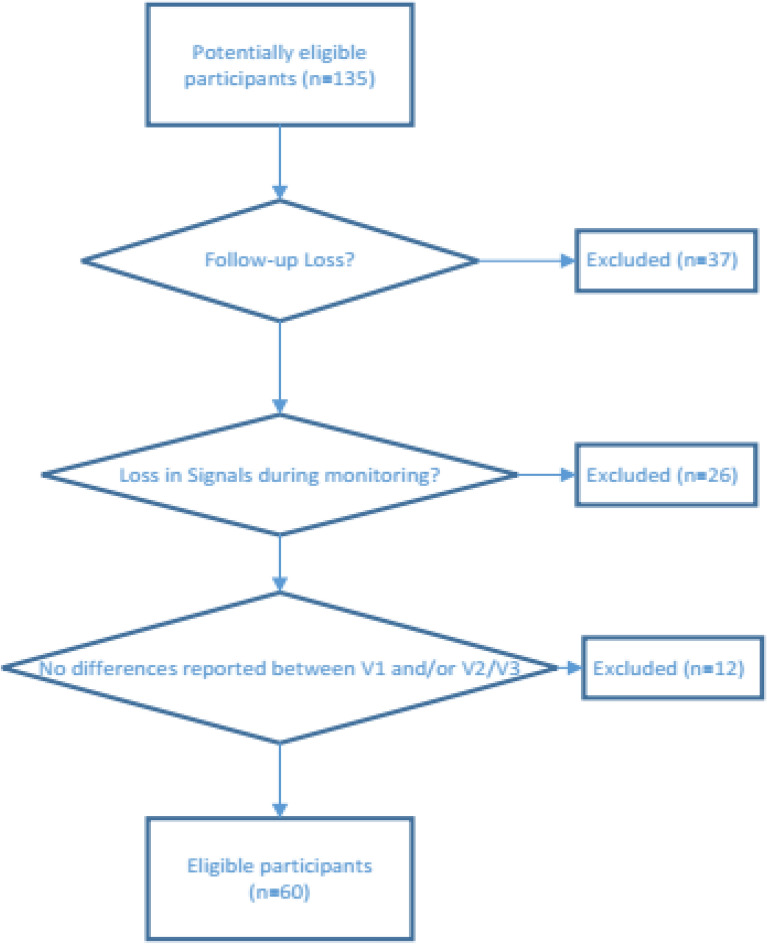
Flow of the study participant selection process.

A total of 135 patients were recruited. After excluding evaluations according to the criteria described above (patients without both V2 and V3 evaluations (home evaluations), signal loss greater than 10% in assessment V1, or V2 and V3; and no improvement of at least one point for dyspnea in the compensated phase), 60 patients were available for inclusion in the analyses. Figure 4 shows the flow of the study participant selection process.

**Figure 5:**
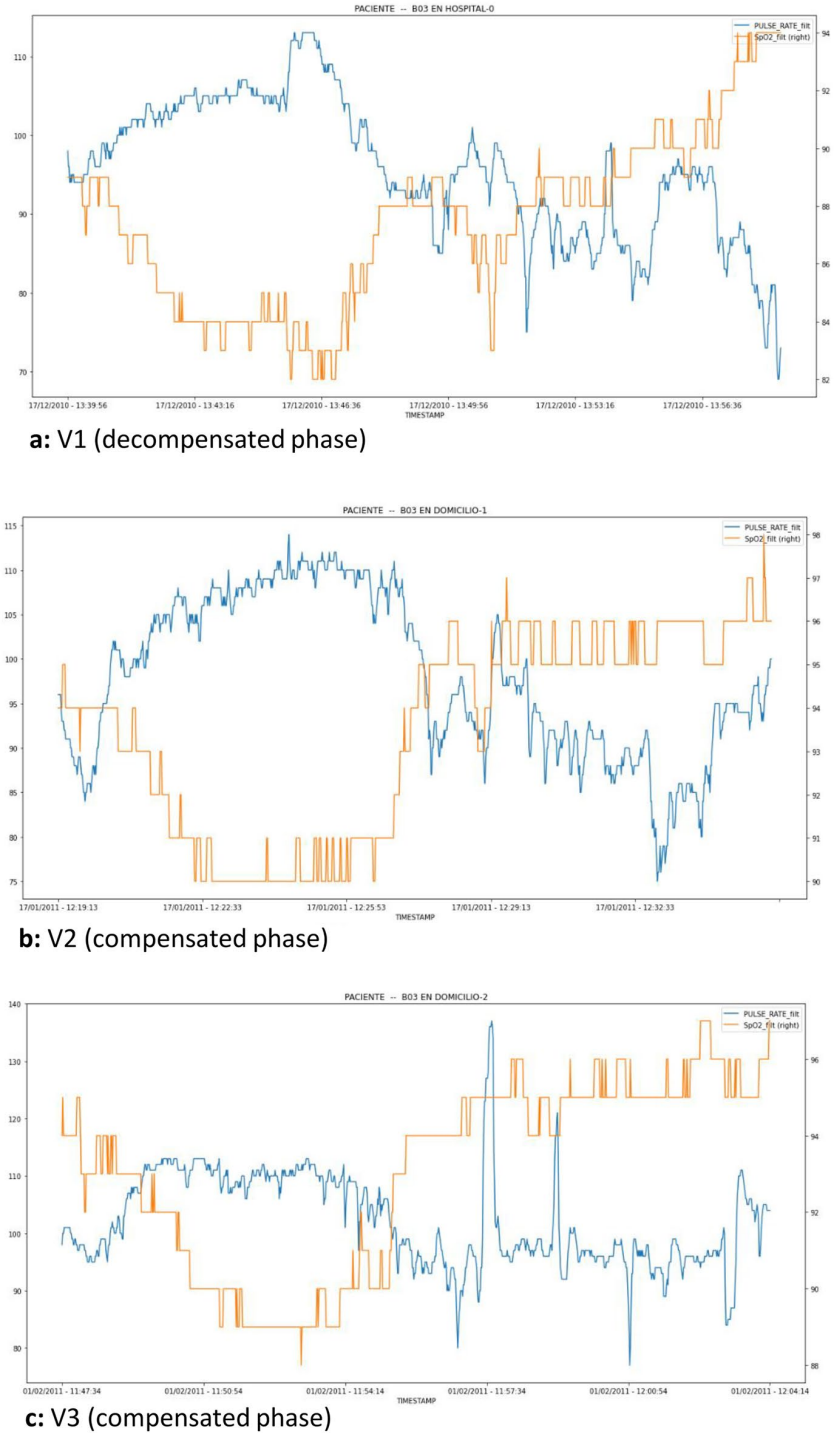
Ox and HR time series for a patient during each evaluation of the trial. The orange and blue lines represent SpO2 and HR, respectively. The HR and SpO2 values correspond to those displayed in the left and right columns, respectively.

Of the 60 patients included, all underwent the hospital evaluation (V1), but not all underwent both home evaluations (V2, V3). Therefore, not all patients included had the six observation units derived from the three planned evaluations (V1, V2, V3). In total, 93 observation units of the “change to decompensation” type (label = 1) and 159 of the “no decompensation” type (label = 0) were obtained. Figure 5 shows an example of the SpO2 and HR values of a patient during the study assessments.

No relevant medical events occurred during the evaluations.

The baseline characteristics of the participants finally selected for model development according to the underlying pathology and the severity of the clinical picture on admission (dyspnea according to the NYHA scale^29^) are shown in Table 1. The means of the walking times (manual recording) were 6.1 min, 5.9 min, and 5.9 min for evaluations V1, V2, and V3, respectively.

**Table 1:** Baseline characteristics of the patients analyzed.

	Decompensated HF	Exacerbated COPD	Decompensation of both pathologies
Total N	22	25	13
Age, years (SD)	78 (8)	72 (8)	75 (11)
Male sex (n, %)	8 (36)	19 (76)	10 (77)
Body mass index (SD)	27 (5)	26 (4)	28 (9)
Type 2 diabetes mellitus (n, %)	9 (41)	6 (24)	5 (38)
Dyslipidemia (n, %)	7 (32)	11 (44)	5 (38)
Active smoking (n, %)	0	7 (28)	1 (8)
Osteoarthritis (n, %)	16 (73)	13 (52)	6 (46)
Mean length of hospital stay in days (SD)	7.6 (4.3)	7.3 (3)	27.4 (43.5)
Previous admissions for HF/COPD, IQR [25, 75]	1.0 IQR [0.0, 2.0]	1.0 IQR [1.0, 2.0]	1.0 IQR [1.0, 3.0]
Number of days prior to discharge at the V1 assessment (SD)	4.9 (16.7)	7.3 (15.4)	4.6 (7)
Dyspnea according to the NYHA scale, IQR [25, 75]	2.0 IQR [2.0, 2.75]	2.0 IQR [1.0, 3.0]	2.0 IQR [1.0, 2.0]
Dyspnea according to the mMRC scale, IQR [25, 75]	3.0 IQR [3.0, 4.0]	3.0 IQR [2.0, 3.0]	4.0 IQR [1.0, 4.0]

### Selected characteristics or predictor variables

Regarding the selection of predictor variables, Table 2 shows the selected characteristics and their descriptions. Of 96 characteristics, 19 were ultimately selected. Using the 3 previously mentioned classification algorithms, we found that the 3 most important predictive characteristics were the following: “meanHRminusOx Recovery”, “meanOxRecovery”, and “meanHRminusOxWalk” (random forest); “PC2-Ox-Recovery”, “PC6-HRminusOx-Walk”, and “stdOxWalk” (gradient boosting classifier); and “meanOxRecovery”, “meanHRminusOx Recovery”, and “stdOxWalk” (LGBM).

**Table 2:** Selected predictor characteristics or variables.

Nomenc**lature	Type	Signal	Phase	Scope
meanHRWalk	Mean	HR	Walk	Temporal
meanHRRecovery			Recovery	
meanOxWalk		Ox	Walk	
meanOxRecovery			Recovery	
meanHRminusOxWalk		HR-Ox	Walk	
meanHRminusOx Recovery			Recovery	
stdHRminusOxWalk	Standard deviation		Walk	
stdOxRecovery		Ox	Recovery	
stdOxWalk			Walk	
frecFirstArmHRRecovery	Frequency of the largest harmonic	HR	Recovery	Frequency
frecSecArmHRminusOxWalk	Frequency of the second largest harmonic	HR-Ox	Walk	
frecSecArmHRminusOxRecovery			Recovery	
frecSecArmHRWalk		HR	Walk	
seconArmOxWalk	Amplitude of the second largest harmonic	Ox		
sumAllArmHRminusOxRecovery	Sum of all harmonics	HR-Ox	Recovery	
sumAllArmOxWalk		Ox	Walk	
PC6-HRminusOx-Walk	Sixth principal component of the FFT	HR-Ox		
PC2-Ox-Recovery	Second principal component of the FFT	Ox	Recovery	
PC4-Ox-Recovery	Fourth principal component of the FFT			

*HR* heart rate, *Ox* oxygen saturation, *PC* principal component, *FFT* fast Fourier transform.

None of the other predictors evaluated (age, sex and baseline disease) provided greater discriminative power to the models.

### Diagnostic algorithms

The diagnostic performance of the algorithms developed according to the technique used is shown in Table 3. The techniques with S and E values above 80% were logistic regression and SVM.

**Table 3:** Diagnostic capacity of the algorithms developed according to the technique used.

Machine learning technique	True positive	False negative	True negative	False positive	Sensitivity *	Specificity *	Accuracy *
Random forest	75	18	138	21	78.3	88.8	83.6
Logistic regression	74	19	129	30	80.8	86.3	83.6
Decision tree	72	21	137	22	78.3	85.8	83.1
Naive Bayes	73	20	142	17	75	90.4	83.1
SVM	77	16	129	30	81.7	85	82.3
LGBM	70	23	132	27	73.3	87.5	80.6
Gradient-boosting classifier	64	29	137	22	69.2	88.3	80.3
KNN	52	41	133	26	53.3	84.2	70.8

*These parameters were obtained from the mean of all patients (since not all had the same number of evaluations, the mean does not necessarily correspond to that obtained from the total true positive, false negative, true negative and false positive data available in the entire sample).

## Discussion

### Main results

The present study reported diagnostic models that achieved a good detection capacity for exacerbation of COPD or HF decompensation (S and E greater than 80%). Although the S and E were slightly lower than those of models in two other studies (Vamos et al.^15^ for HF and Wu et al.^17^ for COPD), we highlighted that the models in our study, unlike these previous models, do not require complex devices such as intradomiciliary sensors or cardiac defibrillators for their implementation in clinical practice. A study that is potentially more comparable to ours in terms of the technology used and the method developed for the algorithms is that of Stehlik et al.^16^. The study reported HF decompensation detection models developed through ML from the monitoring of physiological parameters of 100 patients collected through a cutaneous patch at the thoracic level. The models developed obtained an S of 76 to 88% and an E of 85%, values similar to those of the models in our study. Recently, Morrill et al.^33^ reported diagnostic models of decompensated HF developed with ML techniques with an S of 100% and E of 73% but based on simulated clinical situations and not real patients.

Another important result was that the underlying disease (COPD or HF) did not influence the development or diagnostic performance of the models; thus, to our knowledge, this is the first study to report diagnostic models of decompensation potentially applicable to patients affected by COPD and by HF, which may be relevant given the increasing proportion of patients affected by both pathologies. However, our study can only be considered preliminary at this point because the trial size was modest and the design was not robust enough to confirm that this result is generalizable. Therefore, this result warrants further investigation. As a hypothesis, we propose the coexistence of pathophysiological mechanisms in the decompensation of both diseases, with HR, Ox and their relationship serving as parameters that could represent a relevant common denominator for decompensation of both pathologies. Ox has already shown considerable utility in the detection of acute HF in previous studies^34^ and has been considered the physiological parameter with the greatest discriminative power in COPD^10,35^. In addition, the cutoff point of Ox for the detection of acute HF does not seem to be modified in patients who also have COPD^34^. Our study also proposes HR and its relationship with Ox as parameters of interest in the pathophysiological mechanisms related to decompensation of both diseases because although most of the characteristics chosen for the development of the models (eight of 19) were only related to Ox, four were exclusively related to HR, and the rest (seven of 19) were related to the combination of the two parameters (HR-Ox). In any case, further research should explore this hypothesis.

### Validity

With the methodological approach considered, we believe that none of the selected characteristics or the other potentially predictive variables evaluated were associated with a possible phenomenon of “information leakage” from the outcome variable (compensated or decompensated phase) to the predictor variables (“outcome leakage”)^26^. However, we must recognize a possible “validation leakage”^26^ because we could not use a completely independent sample for validation of the diagnostic models developed (the sample size we had led us to prioritize the development of the models with the maximum available sample), and we must recognize the possibility of some overestimation in the diagnostic performance obtained.

We began this study with a cohort of patients in the decompensated phase. This design allowed us to have sufficient observations for both categories of the outcome variable and to develop and evaluate the models obtained (if we had started with a cohort of stable patients, only a small proportion would have presented with decompensation). In addition, the design allowed each patient to act as his or her own control. Although choosing hospitalization as a reference for the decompensated phase was not ideal because the ultimate goal of these algorithms was to detect clinical decompensation in an earlier phase, the evaluation during hospitalization was performed once the patients were clinically stable and were able to walk at least 30 m, so the hospital evaluation (V1) was actually carried out once the most acute phase of decompensation had passed. In the same way, we did not take admission parameters into account because that moment represents the most severe phase of exacerbation, and the aim of this study was to develop an algorithm based on parameters as similar as possible to an earlier phase of exacerbation.

The time interval between the standard diagnostic method (confirmation of the compensated or decompensated phase by a doctor) and data collection for the development of the diagnostic models was quite short (24–48 h); therefore, we do not believe that considerable changes in the clinical states of the patients occurred between these events to influence the results for the diagnostic performance of the models developed.

In terms of the extrapolation of our results to other populations, the models were designed to detect the most severe exacerbations of the disease (those that prompt hospital admission) and not milder exacerbations (such as those requiring only outpatient management). The inclusion of centers with different levels of complexity in two different geographical areas allowed us to include a sample of patients representing a large part of the clinical spectrum of both pathologies.

### Clinical implications

Pending external validation and demonstration of their efficacy in routine clinical practice, the models developed in this study are designed for implementation in minimally invasive or nondisruptive devices for routine, continuous out-of-hospital monitoring of certain patients. Although the data in this study were collected from a pulse oximeter, various commonly used devices (for example, smartwatches) are capable of continuously monitoring the physiological parameters included in the diagnostic models developed.

### Limitations

In addition to the limitations mentioned in the previous paragraphs, we must recognize the high proportion of valuations missing or excluded from the analysis, which may have adversely influenced the diagnostic performance achieved by the models developed. Thus, we must accept the possibility of a nonnegligible selection bias in the final sample available for analysis. This limitation, along with the modest sample size of our study, prevented us from delving deeper into certain aspects of the analysis and interpretation of the results. We were unable to perform analyses on specific subgroups (e.g., the subgroup of patients with both conditions in a decompensated state), identify phenotypes of the evaluated conditions, or compare the diagnostic performance of the models between different evaluations (e.g., V1–V2 vs. V1–V3).

We also emphasize that the conditions in which the assessments were performed were controlled (a specific protocol of walking and recovery was followed), and evaluations in more “real” conditions are still pending. Finally, although the high proportion of evaluations in the decompensated phase allowed us to enhance model development, this proportion was considerably higher than that in the real world (in usual conditions, most patients are in the compensated phase of their disease); therefore, a compensated/decompensated phase proportion closer to that in the real world should be considered in future studies to avoid a high false-positive rate precluding implementation in clinical practice.

## Conclusions

The diagnostic models developed achieved good diagnostic performance for decompensated HF or COPD exacerbation.

To our knowledge, this study is the first to report diagnostic models of decompensation potentially applicable to both COPD and HF patients. However, these results are preliminary and warrant further investigation to be confirmed.

## Data Availability

The datasets generated and/or analyzed during the current study are available from the corresponding author upon reasonable request.
